# Quantification of Cell-Free HER-2 DNA in Plasma from Breast Cancer Patients: Sensitivity for Detection of Metastatic Recurrence and Gene Amplification

**DOI:** 10.5772/61320

**Published:** 2015-08-31

**Authors:** Patricia Diana Sørensen, Rikke Fredslund Andersen, Niels Pallisgaard, Jonna Skov Madsen, Erik Hugger Jakobsen, Ivan Brandslund

**Affiliations:** 1 Department of Clinical Immunology and Biochemistry, Vejle, Lillebaelt Hospital, Denmark; 2 Institute of Regional Health Research, Faculty of Health Sciences, University of Southern Denmark, Odense, Denmark; 3 Department of Clinical Oncology Vejle, Lillebaelt Hospital, Denmark

**Keywords:** breast cancer, cell-free DNA, gene amplification, HER-2, metastatic recurrence, sensitivity

## Abstract

The purpose of this study was to quantify the free-circulating plasma HER-2 DNA (cfHER-2 DNA) and to assess the ability of analysis to discriminate between patients with primary breast cancer and healthy controls in order to detect metastatic recurrence in comparison with serum HER-2 protein and also HER-2 gene amplification.

The study population consisted of 100 patients with primary breast cancer and 50 healthy female donors. An additional 22 patients with metastases were subsequently included. cfHER-2 DNA was quantified with a quantitative PCR method together with a reference gene.

Results: Using a cut-off of 2.5 for the ratio of the cfHER-2 DNA/reference gene, three (of 15) tissue HER-2-positive patients had a ratio >2.5 prior to the detection of metastatic disease. In the post-metastatic/pre-chemotherapy setting, 11 (of 23) tissue HER-2-positive patients with metastases had a ratio >2.5.

There was no difference between absolute preoperative cfHER-2 DNA values for patients with primary breast cancer and those for healthy controls. There was no difference between cfHER-2 DNA values taken within nine months of development of the metastatic disease and the levels in patients without metastases, but there was a significant difference in the corresponding serum HER-2 protein levels in the tissue HER-2-positive patient group.

Conclusion: Amplified HER-2 DNA can be detected in plasma when using a ratio between cfHER-2 DNA and a reference gene. cfHER-2 DNA could not be used to discriminate between patients with primary breast cancer and healthy controls, and could not predict the development of metastatic disease.

## 1. Introduction

Breast cancer is the most common cancer in women in the Western world. Although significant progress has been made in the adjuvant treatment of primary breast cancer, more than 20% of patients initially diagnosed with regional disease develop systemic relapse later in life [1]. This implies the need for biomarkers for evaluating the effect of treatment after primary therapy and also for early recurrence detection.

The presence of increased free-circulating DNA in serum from cancer patients was described more than 30 years ago [2]. Subsequent studies reported increased levels of free-circulating DNA in plasma/serum from patients affected by colon and lung cancer [3].

The source of the free-circulating DNA is still a debated subject. In healthy people it has been stated to originate from the apoptosis of nucleate cells [4], but some consider it to also originate from active release [5]; [6]. In cancer patients, an increased level is hypothesized to originate from the necrosis of cancer cells, and perhaps also from active release from cancer cells [7]. Another hypothesis is a possible perturbation of the mechanism of DNA clearance from plasma, which is also unconfirmed [5].

Quantification of HER-2 gene-copy number in the circulating cell-free DNA is a novel suggested biomarker to detect possible amplification. Recent studies have investigated its potential for detecting HER-2 amplification in plasma [8] and also for monitoring response to Trastuzumab treatment [9].

The purpose of this study is to quantify the free-circulating plasma HER-2 DNA. For this purpose a quantitative method for detecting plasma HER-2 gene-copy number in the total free-circulating DNA was developed and validated. The study focuses on the ability of the analysis to discriminate between patients with primary breast cancer and healthy controls, as well as to detect metastatic recurrence and establish correlation to tissue HER-2 status and serum HER-2 protein.

## 2. Methods

### 2.1 Study Design and Population

This study was performed on plasma samples from a previous prospective project, which was carried out on breast cancer patients in the period 2004–2009. The monitoring time had a range of 1.1–6.3 years (mean 3.6). The patients were primarily and prospectively monitored with serum HER-2 protein; the total population is described in a previous article [10]. Both plasma and serum samples were available. Serum samples were used to analyse serum HER-2 protein, and plasma samples were used for plasma cell-free DNA.

Of the 841 patients included in the study, 100 consecutive patients who fulfilled the following inclusion and exclusion criteria were selected for the circulating plasma cell-free HER-2 DNA analyses.

Inclusion criteria:

1. Twenty-five tissue HER-2-positive patients known to have elevated serum HER-2 protein levels;
2. Twenty-five tissue HER-2-positive patients known to have normal serum HER-2 protein levels;
3. Twenty-five tissue HER-2-negative patients known to have elevated serum HER-2 protein levels;
4. Twenty-five tissue HER-2-negative patients known to have normal serum HER-2 protein levels.

Exclusion criteria: Patients without available preoperative blood sample and patients with fewer than five blood samples available in the follow-up.

Baseline samples were taken prior to surgery and/or chemotherapy.

An additional 22 patients with metastases (14 tissue HER-2-positive and eight tissue HER-2-negative), who had had a blood sample taken after being diagnosed with metastases and before beginning chemotherapy, were analysed with the purpose of strengthening the results. The results from the additional patients were evaluated together with samples from patients with metastases from the study population.

Fifty healthy female donors (10 aged 25–44 years, 20 aged 45–59 years, and 20 aged 60–75 years) from the control group at the Diabetes Research Bank, Vejle Hospital, served as healthy controls.

The work was approved by the Regional Science Ethics Committee (reg. nr. S-VF-20040101).

### 2.2 Clinical Follow-up

In brief, following surgery for primary breast cancer the patients underwent routine clinical controls at the Oncology Department. Patients with inoperable disease at the time of diagnosis received neoadjuvant treatment prior to surgery.

After surgery, the patients were treated with adjuvant therapy according to the current guidelines from the Danish Breast Cancer Group. Patients with tissue HER-2-positive breast cancer determined by IHC/FISH received Trastuzumab and/or Tykerb. Patients with tissue HER-2-negative breast cancer received adjuvant FEC or EC (Fluorouracil, Epirubicin, Cyclophosphamide or Epirubicin, Cyclophosphamide) and/or Docetaxel. The patients with oestrogen-positive primary tumour received antihormonal treatment.

The patients who presented symptoms of clinical recurrence were further investigated. Metastases were identified by imaging techniques with Computed Tomography/Ultrasound/Magnetic Resonance (CT/UL/MR) and verified by pathological examination, if possible. The levels of free-circulating DNA and serum HER-2 did not contribute to diagnostic decision-making.

### 2.3 Laboratory Methods

#### 2.3.1 Quantification and Validation of Cell-free Plasma HER-2 DNA

1. Samples: The patients had blood tests taken in routine clinical controls at the Oncology Department during the “Serum HER-2 protein project” [10]. Besides serum samples for that project, 3 mL of peripheral blood was also collected in an EDTA tube at each visit. The blood was centrifuged at 2000 × g for 10 minutes within two hours of sampling, and the plasma was divided into two cryotubes and frozen at −80°C until analysis could take place. We used 0.6 mL plasma/patient/visit; a few samples had less volume, between 50 and 550 μL.
2. Purification: DNA was purified with a QIAsymphony robot using the Qiagen Virus/Bacteria Midi Kit (Qiagen, Hilden, Germany). The robot required 1.2 mL volume of each plasma sample; the plasma was therefore supplemented with water up to 1.2 ml, which was taken into account when the results were calculated.
3. Quantitative PCR analyses: Plasma HER-2 gene-copy number and a reference gene-copy number were detected by quantitative real-time PCR using in-house assays. At the same time, we analysed CPP1 (cysteine-rich polycomb-like protein 1), a plant gene [11] that was added to all plasma samples with the purpose of monitoring for loss during the purification process, and B-cell lysis, using a peripheral-blood (PB) assay that detects the rearranged immunoglobulin genes in B-lymphocytes [12]. Results were excluded if they showed signs of B-cell-lysis contamination. The HER-2 gene-copy number was calculated as 10 ^[(y, intercept(gHER2) - meanCt (gHER2)) / slope(gHER2)]^ and the B2M gene-copy number as 10 ^[(y, intercept(gB2M) - meanCt (gB2M)) / slope(gB2M)]“^. The plasma volume was taken into account in the calculation of the results.

Primers and probes for both assays were designed using the Oligo Primer Analysis Software version 7 (Molecular Biology Insights, Colorado, USA).

The following concentrations were used: for the B2M gene – 3 microM forward (F) plus reverse (R) primer and 2 microM probe; for the HER-2 gene – 9 microM forward (F) plus reverse (R) primer and 2 microM probe. Primers were purified using a reverse-phase fast-centrifuge purification method, while the probes were purified using an HPLC method (DNA Technology, Aarhus, Denmark).

Primers and probe for the HER-2 gene:

F-primer: HER2ex26:64U20, 5‘-GCGGTGGGGACCTGACACTA-3’

R-primer: HER2ex26:122L18, 5‘-CCTTCGGAGGGTGCCAGT-3’

Probe: HER2ex26:93F26, Fam-CCCTCTGAAGAGGAGGCCCCCAGGTC-Tamra

Primers and probe for the B2M gene:

F-primer: B2Mex4:972U29, 5‘-TAAAACTTAATGTCTTCCTTTTTTTTCTC-3’

R-primer: B2Mex4:1047L27, 5‘-AAACATTTTCTCAAGGTCAAAAACTTA-3’

Probe: B2Mex4:387L26, Fam-CCTCCATGATGCTGCTTACATGTCTC-Tamra

The reactions were performed on ABI7900HT using 20 μl TaqMan Universal Mastermix (Life Technologies, CA, USA) with primers and probe and 5 μl of purified DNA solution, as follows: initial step 50°C for two minutes and activation step 95°C for 10 minutes, followed by 50 cycles at 95°C for 15 seconds and 60°C for one minute. The results are expressed in copies/mL.

All the analyses were performed in triplicate. The HER-2 gene amplification in the cell-free DNA from plasma was determined by dividing the number of cfHER-2 DNA copies by the number of cfB2M DNA copies.

## 2.4 Validation Process According to the MIQE Guidelines

### 2.4.1 Standard Curves

The levels of plasma cfHER-2 DNA and the reference gene B2M were determined using standard curves calculated with digital PCR, which is the most accurate technique for absolute quantification. Current articles about digital PCR analyses for HER-2 expression in breast-cancer tissue showed good concordance with IHC/FISH [13, 14].

DNA was purified from blood from 50 healthy control subjects and was subsequently pooled. Five microliters of pooled DNA and subsequent five-, 25-, 125-, 625-, 3125-, and 15,625-fold dilutions were all analysed with both digital PCR and quantitative PCR. The results from the digital PCR, counting a colour signal/one copy, were given in copies/microliter. The results from the analysis on the ABI7900HT Real-time PCR System were given in Cq. Standard curves were drawn by matching the Cq values with the copy number from the digital PCR. The slope for the HER-2 standard curve was −3.43, which corresponds to a PCR efficiency (E) of 96% where E = (10 ^ (−1/slope) −1) * 100. The slope for the B2M standard curve was −3.49, corresponding to an efficiency of 93%.

### 2.4.2 Recovery Test

1.2 ml of plasma from a patient with metastatic breast cancer was analysed on ABI7900HT.

The mean values from the triple determination were 20544 (19979, 22674, 18978) HER-2 copies/mL and 5864 (6067, 5231, 6295) B2M copies/mL. Half the portion from the initial plasma samples (600 microliters) was mixed with water and analysed with the following results for the mean value: 8362 HER-2 copies/mL and 2513 B2M copies/mL.

1.2 mL of plasma from the same patient was subsequently diluted 10-fold to produce 10 samples. Each sample was analysed in triplicate and the average was taken as the result of the sample. The mean values were 2153 HER-2 copies/mL (CV=7%) and 824 B2M copies/mL (CV=9%). Each of the 10-fold dilutions was again diluted 10 times to produce 10 samples with the following mean values: 230 HER-2 copies/mL (CV=9%) and 74 B2M copies/mL (CV=9%). Each of the 100-fold dilutions was again diluted 10 times to produce 10 samples with the following mean values: 21 HER-2 copies/mL (CV=19%) and seven B2M copies/mL (CV=25%).

### 2.4.3 Quality Control, Reproducibility

Genomic DNA from healthy donors was used as control material and was analysed with each master-mix. CV over an approx. six-month period was 15% for HER-2 copy detection and 14% for B2M copy detection.

### 2.4.4 Repeatability

Repeatability in the normal area was determined by performing repeated measurements on the same sample. 20 mL of whole blood was collected from two healthy donors. The plasma obtained from each donor was divided into 10 portions and the DNA purified and analysed on ABI7900HT. The first donor had a mean of 1901 HER-2 copies/mL (CV=20%) and a mean of 1621 B2M copies/mL (CV=23%). The results from the second donor showed a mean of 2490 HER-2 copies/mL (CV=22%) and a mean of B2M copies/mL of 2280 (CV=17%).

### 2.4.5 Carry-over

DNA purification was done using the QIAsymphony robot, which placed water samples between plasma samples in order to detect possible cross-contamination. No cross-contamination was detected. Water samples were also used as a negative control for each master-mix – Non Template Control (NTC). No positive NTCs were detected.

### 2.4.6 Reference Range

Reference range was determined using values from healthy controls. Plasma from 50 healthy controls was analysed for HER-2, B2M, CPP1 and PB. Seven controls were excluded because of B-cell contamination. The remaining 43 healthy controls had values between 1591 and 6252 HER-2 copies/mL (mean 3556) and 97.5 percentile 6244 HER-2 copies/mL (95%CI 5637 –6252). The values for the reference gene B2M were between 1377 and 6019 B2M copies/mL (mean 2923) and 97.5 percentile 5975 B2M copies/mL (95%CI 4626–6019).

### 2.4.7 Analytical Sensitivity

The detection limit was determined according to a dilution experiment where 100,000 HER-2/B2M copies from a normal cell-line MCF10A were added to 100 microliters of water. After analyses, repeated dilutions were performed (first two-fold, then four-fold then 10-fold, 100-fold, 1000-fold and 10,000-fold). The mean value for the lowest detection limit was 40 HER-2 copies/mL (range 20–80 copies/mL; CV=42%) and 20 B2M copies/mL (range 0–20 copies/mL; CV=42%).

### 2.4.8 Analytical Specificity

**Figure 1.**
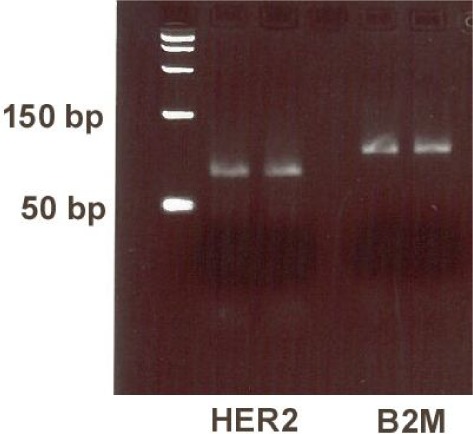
In gel: HER-2 gene PCR product corresponding to 76 bp and B2M gene PCR product corresponding to 102 bp

The primers and probe ensured analytical specificity. A blast search was performed and there were no other predicted amplified domains. HER-2 and B2M gene amplification products were both run in agarose gel and each showed one band corresponding to the predicted amplicon length (Fig. 1).

### 2.4.9 Method Comparison

Page et al. used a similar method in their article about the detection of HER-2 amplification in cfDNA from breast cancer patients. However, they used other primers for targeting the HER-2 gene, another reference gene (GAPDH), and a delta Cq for determining the HER-2 gene amplification.

## 2.5 Serum HER-2 Protein

Serum HER-2 was previously analysed using the ADVIA Centaur assay, which is an automated sandwich immunoassay that uses direct chemiluminescent technology and two monoclonal antibodies specific to unique epitopes on the extracellular domain of the HER-2 receptor [15]. Serum HER-2 values above 15 μg/L were considered positive. Analytical CV% in the period was determined by commercial and in-house controls at 8, 14 and 113 μg/L; the values were 6.6, 4.6 and 4.8, respectively.

## 2.6 Pathological Analyses with IHC/FISH

Tissue HER-2 was routinely assessed with IHC and FISH. IHC 3+ or IHC 2+ and FISH>2.0 were considered positive. IHC and FISH were performed on formalin-fixed paraffin-embedded breast-cancer tissue. IHC was analysed using the Hercep Test (DakoCytomation, Glostrup, Denmark) and FISH was analysed with the HER-2 FISH pharmDx kit (DakoCytomation).

### 2.6.1 Statistical Analysis

Statistical analyses were performed on STATA IC 11. A Mann-Whitney test was used to compare two patient groups. A Fisher-exact or chi2 test were used to compare data from 2×2 tables.

## 3. Results

The characteristics of the follow-up patients are presented in Table 1. In total, 1,142 analyses were performed on plasma from the followed patients, with each patient having a minimum of five samples. Eighty-five patient samples and seven samples from the healthy controls that were positive for PB (a sign of B-cell-lysis contamination) were excluded from the data evaluation. Thirteen patient samples with haemolysis were also excluded.

**Table 1.** Patient population characteristics: 100 follow-up patients

Age, years	
<=40	1
41-50	19
51-60	31
61-70	33
>=70	16
Tissue HER-2 status	
Positive	50
Negative	50
Tumour size at diagnosis (mm)	
T1	40
T2	52
T3	3
T4	2
Tx	3
No. of positive nodes at diagnosis	
N1	83
N2	12
N3	0
Nx	5
Metastases status at diagnosis	
M0	100
Histological type	
Invasive ductal carcinoma	88
Invasive lobular carcinoma	6
Other	5
Unknown	1
Oestrogen receptor status	
Positive	69
Negative	31
Progesterone receptor status	
Positive	53
Negative	44
Unknown	3

Twenty-five follow-up patients with initial primary breast cancer developed metastatic disease in the monitoring period. The results from the follow-up patients who developed metastatic disease were analysed together with the results from the additional patients with metastases.

### 3.1 Preoperative Plasma/serum Samples from Tissue HER-2-positive Patients vs. Healthy Controls

**Figure 2.**
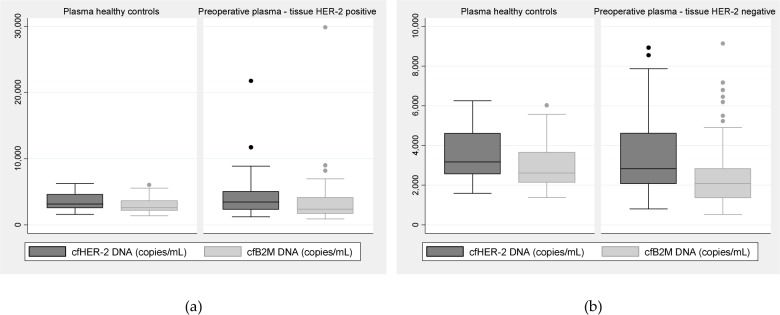
(a) Preoperative plasma cfHER-2 DNA and cfB2M DNA levels from tissue HER-2-positive patients compared with levels from healthy controls. (b) Preoperative plasma cfHER-2 DNA and cfB2M DNA from tissue HER-2-negative patients compared with levels from healthy controls.

Of the 50 preoperative plasma samples from patients with tissue HER-2-positive primary tumour, seven samples were excluded because of B-cell-lysis contamination. There was no significant difference between preoperative plasma cfHER-2 DNA or cfB2M DNA from tissue HER-2-positive patients and healthy controls (Suppl. Table 1, Figure 2A). There was no correlation between preoperative plasma cfHER-2 DNA and the corresponding serum HER-2 protein (range 6-52, mean 14.8, median 13 μg/L) in the tissue HER-2-positive patient group (p=0.14).

### 3.2 Preoperative Plasma/serum Samples from Tissue HER-2-negative Patients vs. Healthy Controls

Of the 50 preoperative plasma samples from patients with tissue HER-2-negative primary tumour, four samples were excluded because of B-cell-lysis contamination. There was no significant difference between preoperative plasma cfHER-2 DNA from tissue HER-2-negative patients and healthy controls (Suppl. Table 1). The preoperative plasma cfB2M DNA values from tissue HER-2-negative patients were lower than the cfB2M DNA values from healthy controls (p=0.02) (Figure 2B). There was no correlation between preoperative plasma cfHER-2 DNA and the corresponding serum HER-2 protein (range 6–21, mean 12, median 11.75 μg/L) in the tissue HER-2-negative patient group (p=0.96).

### 3.3 Ability of cfHER-2 DNA to Detect Metastatic Recurrence Compared with serum HER-2 Protein

#### 3.3.1 Samples Taken before Detection of Metastatic Disease Compared to Patients without Metastasis (Tissue-positive Patients)

Of the 50 tissue HER-2-positive patients, 18 patients developed metastatic disease in the monitoring period, while 32 remained clinically relapse-free. The plasma/serum sample from patients with metastases was taken within nine months of detection of symptomatic metastases. For the patients without metastatic disease, the highest value taken in a period without chemotherapy was chosen. Three plasma samples from patients with metastasis were excluded because of B-cell contamination.

There was no significant difference between the plasma cfHER-2 DNA or cfB2M DNA values in patients before the development of metastatic disease and those in patients without metastasis (Suppl. Table 2). There was a significant difference between the corresponding serum HER-2 levels taken before the development of metastatic disease (range 4–1729, mean 162, median 22.3 μg/L) and serum HER-2 levels in patients without metastasis (range 5–18, mean 10, median 10.1 μg/L) (p=0.0003). In our previous work [10], we found a good correlation between increased serum HER-2 values and the development of metastatic disease in tissue-positive patients.

**Table 2.** Number of tissue HER-2-positive patients with elevated or normal serum HER-2, cfHER-2 DNA and cfB2M DNA levels prior to metastases detection

Serum HER-2 prior to metastases detection (tissue-positive)	With metastases	Without metastases	Total
Elevated	8	3	11
Normal	7	29	36
Total	15	32	47
CfHER-2 DNA prior to metastases detection (tissue-positive)	With metastases	Without metastases	Total
Elevated	8	9	17
Normal	7	23	30
Total	15	32	47
CfB2M DNA prior to metastases detection (tissue-positive)	With metastases	Without metastases	Total
Elevated	6	9	15
Normal	9	23	32
Total	15	32	47

We have also examined the ability of cfHER-2 DNA and cfB2M DNA to detect metastatic recurrence compared to serum HER-2 protein using cut-offs obtained from healthy controls, which were 6244 HER-2 copies/mL for cfHER-2 DNA and 5975 B2M copies/mL for the reference gene B2M. For the serum HER-2 protein, we used the known cut-off of 15 microgram/L. The results in the tissue HER-2-positive group showed a significant difference between the metastatic and non-metastatic group in the serum HER-2 protein values (p=0.002 with a sensitivity of 53% [95%CI 27–77] and a specificity of 90% [95%CI 73–97]) but no significant difference for cfHER-2 DNA (p= 0.09 with a sensitivity of 53% [95%CI 27–77] and a specificity of 71% [95%CI 53–85]) or for cfB2M DNA (p=0.41 with a sensitivity of 40% [95%CI 17–67] and a specificity of 71% [95%CI 53–85]) (Table 2).

#### 3.3.2 Samples Taken before Detection of Metastatic Disease Compared with Patients without Metastasis (Tissue-negative Patients)

Of the 50 tissue HER-2-negative patients, 10 patients developed metastatic disease in the monitoring period, while 40 remained clinical relapse-free. The plasma/serum sample from patients with metastasis was taken within nine months of the detection of symptomatic metastasis. There was no significant difference between plasma cfHER-2 DNA or cfB2M CNA in patients before the development of metastatic disease and patients without metastasis (Suppl. Table 3). There was no significant difference between the corresponding serum HER-2 levels taken before the development of metastatic disease (range 10.3–36.7, mean 17, median 14 μg/L) and serum HER-2 from patients without metastasis (range 7.3-23.4, mean 13, median 12 μg/L) (p=0.13). In our previous article [10], we found no correlation between increased serum HER-2 values and the development of metastatic disease in tissue-negative patients. Using the previously specified cut-offs, the results in the tissue HER-2-negative group showed no difference between the metastatic and non-metastatic group for either serum HER-2 protein (p=0.3 sensitivity of 40% [95%CI 13–72], specificity 72% [95%CI 55–84]), cfHER-2 DNA (p= 0.1, sensitivity of 40% [95%CI 13–72], specificity of 85% [95%CI 69–93]) or cfB2M DNA (p=0.1 with a sensitivity of 30% [95%CI 8–64], and a specificity of 90% [95%CI 75–96]) (Table 3).

**Table 3.** Number of tissue HER-2-negative patients with elevated or normal serum HER-2, cfHER-2 DNA and cfB2M DNA levels prior to metastases detection

Serum HER-2 prior to metastases detection (tissue-negative)	With metastases	Without metastases	Total
Elevated	4	11	15
Normal	6	29	35
Total	10	40	50
CfHER-2 DNA prior to metastases detection (tissue-negative)	With metastases	Without metastases	Total
Elevated	4	6	10
Normal	6	34	40
Total	10	40	50
CfB2M DNA prior to metastases detection (tissue-negative)	With metastases	Without metastases	Total
Elevated	3	4	7
Normal	7	36	43
Total	10	40	50

### 3.4 Detection of HER-2 gene Amplification in Plasma cfDNA

**Figure 3.**
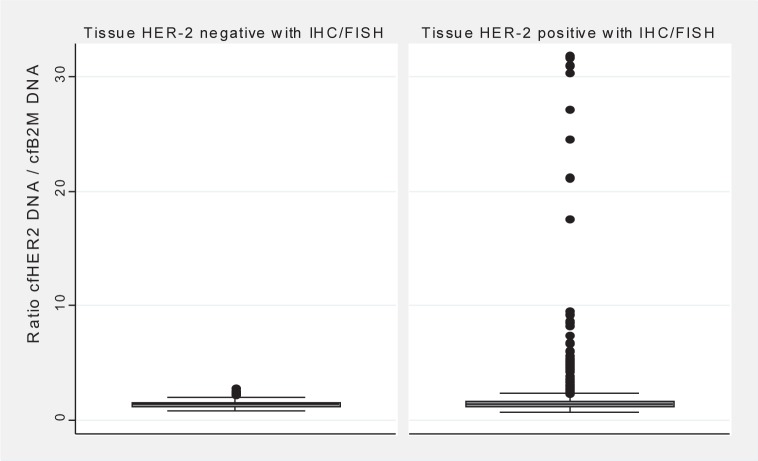
Ratio levels of cfHER-2 DNA/cfB2M DNA in tissue HER-2-negative patients (n=454) compared to tissue HER-2-positive patients (n=590)

cfHER-2 DNA/cfB2M DNA ratio levels had a range of 0.69–31.88 (mean 1.95, median 1.38) in 590 follow-up samples from 50 tissue HER-2-positive patients. Samples from the tissue HER-2-negative patients (454) had a ratio-level range of 0.75–2.76 (mean 1.38, median 1.34). There was a significant difference between the ratio levels of the tissue-positive patients compared with those of the tissue-negative patients (p=0.03) (Figure 3).

If a cut-off of 2.5 (which is 99 percentiles from the tissue HER-2-negative values) was chosen, 46 (8%) samples from the tissue HER-2-positive group had a ratio >2.5 and four (0.9%) samples from the tissue-negative group had a ratio >2.5.

### 3.5 Ratio Levels from Preoperative Samples

In the preoperative setting, there was no significant difference between the ratio levels of the tissue HER-2-positive patients compared with those of the tissue HER-2-negative patients or the healthy controls (Suppl. Table 4). One preoperative sample from the tissue HER-2-positive group had ratio >2.5 and the same was observed for the tissue-negative group.

**Table 4.** Ratio between cfHER-2 DNA and cfB2M DNA prior to metastases detection

	Ratio HER-2/B2M	Ratio	p-value	p-value
**Tissue HER-2-positive patients (N=15)**	Range	0.92–6.77	0.34	0.0001
	Mean	2.26		
	Median	1.524		
**Tissue HER-2-negative patients (N=10)**	Range	0.98–2.23	1.0	0.001
	Mean	1.55		
	Median	1.520		
**Healthy controls (N=47)**	Range	0.08–1.59		1.0
	Mean	1.23		
	Median	1.25		

### 3.6 Ratio Levels before Detection of Metastatic Disease

Ratio levels from samples taken within nine months before the detection of metastatic disease in 15 tissue-positive patients were compared with ratio levels from 10 tissue-negative patients, but the results showed no statistically significant difference between the two groups (Table 4). When the results were compared with the ratio levels from healthy controls, there was a statistically significant difference between the ratio levels of healthy controls and the ratio levels of tissue-positive patients (p=0.0001) but also between the ratio levels of healthy controls and the ratio levels of tissue-negative patients (p=0.001). Three (of 15) tissue HER-2-positive patients had ratio >2.5 prior to metastases detection. Regarding the tissue-negative group, none of the patients (out of 10) had ratio >2.5 prior to metastases detection.

### 3.7 Ratio Levels after Detection of Metastatic Disease

We also examined the ratio levels after the diagnosis of metastatic disease prior to the start of palliative chemotherapy both in the available samples from the study population (nine tissue HER-2-positive and eight tissue HER-2-negative patients) and in the extra samples (14 tissue HER-2-positive and eight tissue HER-2-negative patients), in total 39 patients: 23 tissue HER-2-positive and 16 tissue HER-2-negative.

In the post-metastatic/pre-chemotherapy setting, there was a significant difference between the ratio levels in the tissue HER-2-positive group and those in the tissue-negative group (p=0.01) (Table 5). In the tissue HER-2-positive group, 11 patients had ratio >2.5 while in the tissue-negative group none had ratio >2.5.

**Table 5.** Ratio between cfHER-2 DNA and cfB2M DNA after metastases detection

	Ratio HER-2/B2M	Ratio	p-value
**Tissue HER-2-positive patients (N=23)**	Range	0.9–10.7	0.01
	Mean	3.55	
	Median	2.4	
**Tissue HER-2-negative patients (N=16)**	Range	0.9–2.23	1.0
	Mean	1.42	
	Median	1.4	

## 4. Discussion

The results gained in our study showed no difference between the absolute preoperative cfHER-2 DNA values for patients with primary breast cancer, regardless of tissue HER-2 status, and absolute cfHER-2 DNA values for healthy controls. The explanation here could be that the tumour is localised, and that very little tumour DNA is released into circulation. The same was observed for the levels of the reference gene B2M, which was used to express the total level of cfDNA in the tissue HER-2-positive group.

The results published in the literature on the levels of cfDNA in patients with primary breast cancer are contradictory. Most studies show a higher level of total cfDNA in plasma or serum from patients with primary breast cancer compared with healthy controls [16][Bibr bibr18-61320]–[19], but others find no difference between the two groups [20, 21]. Holdenrieder et al. [21] found no difference in plasma cfDNA concentrations between patients with benign diseases and patients with various types of primary cancer including breast cancer.

One recent study [20] has investigated HER-2 gene amplification in plasma cfDNA and the total cfDNA in patients with primary breast cancer during neoadjuvant chemotherapy. The authors found no difference in the baseline total plasma cell-free DNA or HER-2 gene-copy number compared to healthy controls, and no difference in baseline plasma HER-2 gene-copy number between tissue HER-2-positive and -negative patients; nor were they able to detect gene amplification in plasma in the tissue HER-2-positive patient group with primary breast cancer.

The discrepancies between cfDNA analysis results could be explained by different sample material (serum or plasma), different assays, or B-cell-lysis contamination. A recent study performed at our department by Andersen et al. [22] found plasma more suitable than serum for cfDNA analysis. B-cell-lysis contamination may cause false positive values. In the present study, a control assay was used which detects rearranged genes that are only present in lymphocytes (PB assay), and in this way it was possible to exclude contaminated samples. Furthermore, not all the performed studies are reported according to the MIQE guidelines.

Regarding the patients who developed metastatic disease in the present study, no difference was found between cfHER-2 DNA or cfB2M DNA levels taken before (within nine months of) the development of metastatic disease and those taken for patients without metastases. Neither specific cfHER-2 DNA nor the total cfDNA (reference gene B2M) could predict the development of metastatic disease. On the other hand, in the tissue HER-2-positive patient group, there was a significant difference between the corresponding serum HER-2 protein levels before recurrence developed and those for patients without recurrence. The difference in the tissue-negative group was not significant. This agrees with the previous work on serum HER-2 protein [10], where a high correlation was found between the increase in serum HER-2 and metastatic recurrence for tissue-positive patients, though the correlation was not significant for the tissue-negative group after adjustment for confounders with a logistic regression analysis.

Regarding the HER-2 gene amplification in plasma, the ratio between cfHER-2 DNA and the reference gene cfB2M DNA was significantly higher in the tissue HER-2-positive group compared with the tissue-negative group when all the patient values were used. The values after the development of metastasis could also be useful in a clinical setting in order to differentiate between HER-2-positive and -negative disease, since it is known that amplification status is not always the same in metastatic tissue as in the primary tumour. After the development of metastatic disease and prior to start of treatment, there was a significant difference between the ratio levels in the tissue HER-2-positive group and those in the tissue-negative group.

Others have examined the same issue. Page et al. [8] found amplification in cfDNA in eight out of 68 patients following treatment for primary breast cancer and also in five out 30 patients with metastatic disease, but not in 22 patients with primary breast cancer. Bechmann et al. [20] included 15 patients with diagnosed metastatic disease (nine tissue HER-2-positive and six tissue HER-2-negative) in their study and found significantly higher plasma HER-2 amplification in the tissue-positive group compared to healthy controls, but not for the tissue-negative group. Sorensen et al. [9] found plasma HER-2 amplification in 50% (14 of 28) of patients with tissue HER-2-positive metastatic breast cancer using a cut-off value of 2xSD above the mean of the controls.

## 5. Conclusion

We conclude that in our population, neither absolute values of specific cfHER-2 DNA nor the total cfDNA (reference gene B2M) could discriminate between patients with primary breast cancer and healthy controls, or predict the development of metastatic disease. Amplified HER-2 DNA can be detected in plasma, mostly in the tissue-positive group and after the diagnosis of metastatic disease. More studies are needed in order to validate the usefulness of detection of amplified cfHER-2 DNA in plasma from breast cancer patients. Improvement of the method of detection of cell-free DNA could facilitate interpretation of results and increase both the sensitivity and the specificity of the analysis. In our population, with the current methods, serum HER-2 protein seems more sensitive and reliable in detecting metastatic recurrence than cell-free DNA.

## 6. Abbreviations

CA 15-3: cancer antigen 15-3; CEA: carcinoembryonic antigen; HER-2: human epidermal growth factor receptor 2; IHC: immunohistochemistry; FISH: fluorescence in-situ hybridization; FEC: Fluorouracil, Epirubicin, Cyclophosphamide; CPP1: cysteine-rich polycomb-like protein 1; PB: peripheral blood; B2M: beta-2 microglobulin; HPLC: high-performance liquid chromatography; Cq: cycle threshold; GAPDH: glyceraldehyde 3-phosphate dehydrogenase; NTC: Non Template Control

## 7. Conflict of interests

Ivan Brandslund has received honoraria from Siemens Denmark for two lectures about serum HER-2 protein.

## References

[1] DBCG Danish Breast Cancer Cooperative Group guidelines. 2011 Jun 15.

[2] Leon SA Shapiro B Sklaroff DM Yaros MJ. Free DNA in the serum of cancer patients and the effect of therapy. Cancer Res 1977;37:646–50. 837366

[3] Anker P Mulcahy H Chen XQ Stroun M. Detection of circulating tumour DNA in the blood (plasma/serum) of cancer patients. Cancer Metastasis Rev 1999;18:65–73. 10505546 10.1023/a:1006260319913

[4] Pathak AK Bhutani M Kumar S Mohan A Guleria R. Circulating cell-free DNA in plasma/serum of lung cancer patients as a potential screening and prognostic tool. Clin Chem 2006;52:1833–42. 16423903 10.1373/clinchem.2005.062893

[5] van der Vaart M and Pretorius PJ. The origin of circulating free DNA. Clin Chem 2007;53:2215. 18267930 10.1373/clinchem.2007.092734

[6] van der Vaart M and Pretorius PJ. Characterization of circulating DNA in healthy human plasma. Clin Chim Acta 2008;395:186. 18519034 10.1016/j.cca.2008.05.006

[7] Pinzani P Salvianti F Pazzagli M Orlando C. Circulating nucleic acids in cancer and pregnancy. Methods 2010;50:302–7. 20146940 10.1016/j.ymeth.2010.02.004

[8] Page K Hava N Ward B Brown J Guttery DS Ruangpratheep C Blighe K Sharma A Walker RA Coombes RC Shaw JA. Detection of HER2 amplification in circulating free DNA in patients with breast cancer. Br J Cancer 2011;104:1342–8. 21427727 10.1038/bjc.2011.89 PMC3078598

[9] Sorensen BS Mortensen LS Andersen J Nexo E. Circulating HER2 DNA after trastuzumab treatment predicts survival and response in breast cancer. Anticancer Res 2010;30:2463–8. 20651409

[10] Sorensen PD Jakobsen EH Madsen JS Petersen EB Andersen RF Ostergaard B Brandslund I. Serum HER-2: sensitivity, specificity, and predictive values for detecting metastatic recurrence in breast cancer patients. J Cancer Res Clin Oncol 2013;139:1005–13. 23483255 10.1007/s00432-013-1411-7 PMC11824405

[11] Cvitanich C Pallisgaard N Nielsen KA Hansen AC Larsen K Pihakaski-Maunsbach K Marcker KA Jensen EO. CPP1, a DNA-binding protein involved in the expression of a soybean leghemoglobin c3 gene. Proc Natl Acad Sci USA 2000;97:8163–8. 10859345 10.1073/pnas.090468497 PMC16687

[12] Pallisgaard N Spindler KL Andersen RF Brandslund I Jakobsen A. Controls to validate plasma samples for cell free DNA quantification. Clin Chim Acta 2015;446:141–6. 25896958 10.1016/j.cca.2015.04.015

[13] Belgrader P Tanner SC Regan JF Koehler R Hindson BJ Brown AS. Droplet digital PCR measurement of HER2 copy number alteration in formalin-fixed paraffin-embedded breast carcinoma tissue. Clin Chem 2013;59:991–4. 23358413 10.1373/clinchem.2012.197855

[14] Heredia NJ Belgrader P Wang S Koehler R Regan J Cosman AM Saxonov S Hindson B Tanner SC Brown AS Karlin-Neumann G. Droplet Digital PCR quantitation of HER2 expression in FFPE breast cancer samples. Methods 2013;59:S20–S23. 23036330 10.1016/j.ymeth.2012.09.012

[15] Luftner D Cheli C Mickelson K Sampson E Possinger K. ADVIA Centaur HER-2/neu shows value in monitoring patients with metastatic breast cancer. Int J Biol Markers 2004;19:175–82. 15503818 10.1177/172460080401900301

[16] Gal S Fidler C Lo YM Taylor M Han C Moore J Harris AL Wainscoat JS. Quantitation of circulating DNA in the serum of breast cancer patients by real-time PCR. Br J Cancer 2004;90:1211–5. 15026803 10.1038/sj.bjc.6601609 PMC2409649

[17] Kohler C Radpour R Barekati Z Asadollahi R Bitzer J Wight E Burki N Diesch C Holzgreve W Zhong XY. Levels of plasma circulating cell free nuclear and mitochondrial DNA as potential biomarkers for breast tumors. Mol Cancer 2009;8:105. 19922604 10.1186/1476-4598-8-105 PMC2780981

[bibr18-61320] Zanetti-Dallenbach R Wight E Fan AX Lapaire O Hahn S Holzgreve W Zhong XY. Positive correlation of cell-free DNA in plasma/serum in patients with malignant and benign breast disease. Anticancer Res 2008;28:921–5. 18507037

[19] Zhong XY Ladewig A Schmid S Wight E Hahn S Holzgreve W. Elevated level of cell-free plasma DNA is associated with breast cancer. Arch Gynecol Obstet 2007;276:327–31. 17431649 10.1007/s00404-007-0345-1

[20] Bechmann T Andersen RF Pallisgaard N Madsen JS Maae E Jakobsen EH Bak Jylling AM Steffensen KD Jakobsen A. Plasma HER2 amplification in cell-free DNA during neoadjuvant chemotherapy in breast cancer. J Cancer Res Clin Oncol 2013;139:995–1003. 23479212 10.1007/s00432-013-1413-5 PMC11824758

[21] Holdenrieder S Burges A Reich O Spelsberg FW Stieber P. DNA integrity in plasma and serum of patients with malignant and benign diseases. Ann N Y Acad Sci 2008;1137:162–70. 18837942 10.1196/annals.1448.013

[22] Pallisgaard N Spindler KL Andersen RF Brandslund I Jakobsen A. Controls to validate plasma samples for cell free DNA quantification. Clin Chim Acta, 446 (2015), p. 141–146). 25896958 10.1016/j.cca.2015.04.015

